# Occurrence of 1,3-Diphenylguanidine, 1,3-Di-*o*-tolylguanidine, and 1,2,3-Triphenylguanidine in Indoor Dust from 11 Countries: Implications for Human Exposure

**DOI:** 10.1021/acs.est.3c00836

**Published:** 2023-04-03

**Authors:** Zhong-Min Li, Kurunthachalam Kannan

**Affiliations:** † Department of Pediatrics, New York University Grossman School of Medicine, New York, New York 10016, United States; ‡ Department of Environmental Medicine, New York University Grossman School of Medicine, New York, New York 10016, United States

**Keywords:** 1,3-diphenylguanidine, 1,3-di-*o*-tolylguanidine, 1,2,3-triphenylguanidine, indoor dust, rubber additive, human exposure

## Abstract

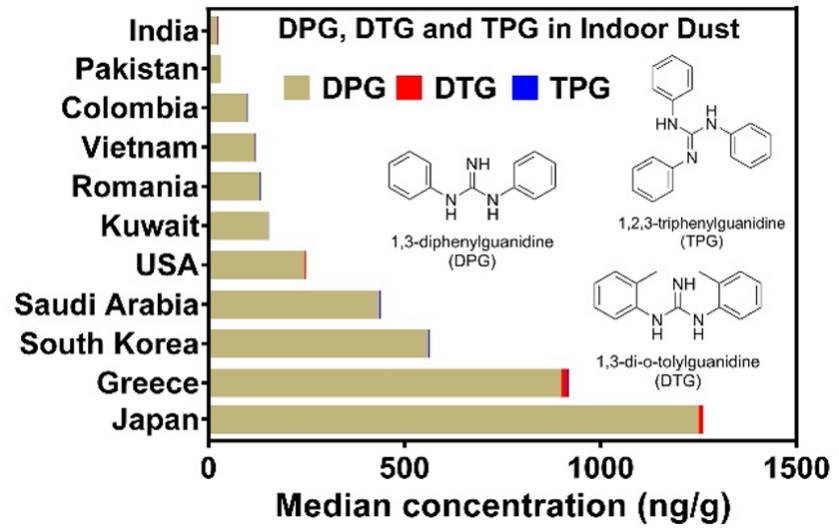

1,3-Diphenylguanidine (DPG), 1,3-di-*o*-tolylguanidine (DTG), and 1,2,3-triphenylguanidine (TPG) are synthetic chemicals widely used in rubber and other polymers. Nevertheless, limited information is available on their occurrence in indoor dust. We measured these chemicals in 332 dust samples collected from 11 countries. DPG, DTG, and TPG were found in 100%, 62%, and 76% of the house dust samples, at median concentrations of 140, 2.3, and 0.9 ng/g, respectively. The sum concentrations of DPG and its analogues varied among the countries in the following decreasing order: Japan (median: 1300 ng/g) > Greece (940) > South Korea (560) > Saudi Arabia (440) > the United States (250) > Kuwait (160) > Romania (140) > Vietnam (120) > Colombia (100) > Pakistan (33) > India (26). DPG accounted for ≥87% of the sum concentrations of the three compounds in all countries. DPG, DTG, and TPG exhibited significant correlations (*r*: 0.35–0.73; *p* < 0.001). Elevated concentrations of DPG were found in dust from certain microenvironments (e.g., offices and cars). Human exposure to DPG through dust ingestion were in the ranges 0.07–4.40, 0.09–5.20, 0.03–1.70, 0.02–1.04, and 0.01–0.87 ng/kg body weight (BW)/day for infants, toddlers, children, teenagers, and adults, respectively.

## Abstract 2

1,3-Diphenylguanidine (DPG), 1,3-di-*o*-tolylguanidine (DTG), and 1,2,3-triphenylguanidine (TPG) are widely present in indoor dust samples from 11 countries.

## 1 Introduction

1,3-Diphenylguanidine (DPG), 1,3-di-*o*-tolylguanidine (DTG), and 1,2,3-triphenylguanidine (TPG) (Table 1) are synthetic chemicals used in the vulcanization of rubber and other polymers,^1^ which are employed in the production of tires, building materials, flooring, furniture, toys, leather products, foot-wear, electronic equipment, and synthetic rubber gloves.^2−6^ The respective annual production of DPG and DTG in the United States in 2015 was 454–9070 and 94 tons (https://chemview.epa.gov/chemview/) and that in Europe in 2018 was 1000–10000 and 100–1000 tons.^2,5^ DPG, DTG, and TPG can be leached from rubber products into the environment, resulting in potential human exposure.

**Table 1:**
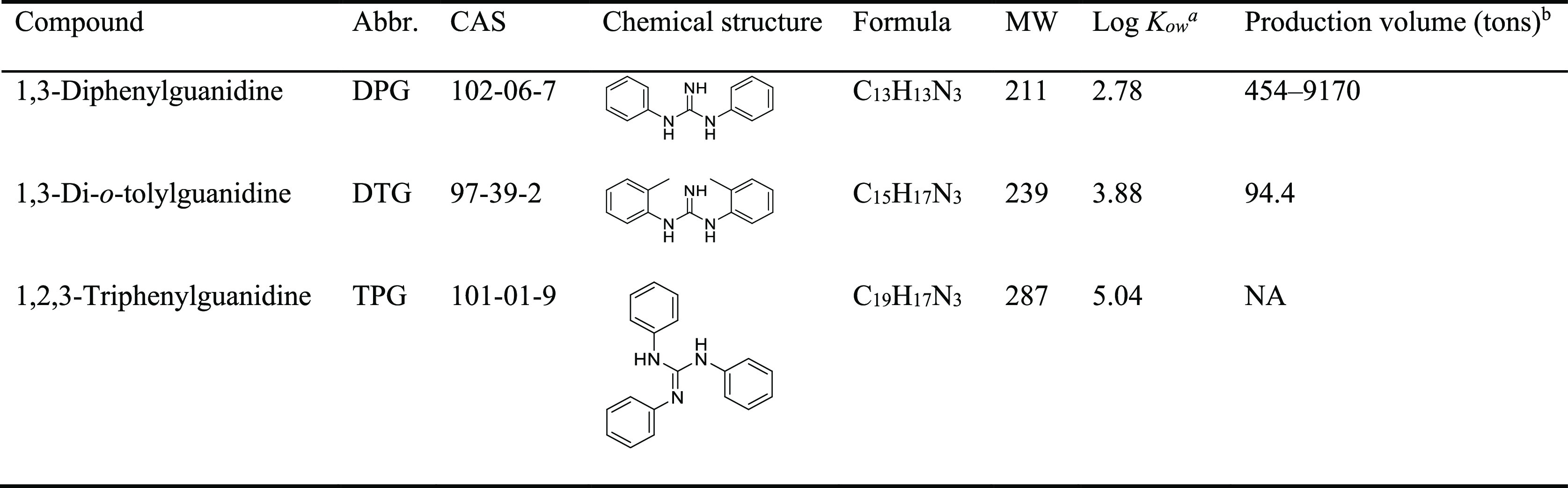
Names, Abbreviations, Chemical Structures, log *K*_ow_ and Production Volumes of the Analytes Investigated in This Study^a^^,^^b^^,^^c^

a Data obtained from ChemSpider: http://www.chemspider.com/.

b The annual national aggregate production volumes in the United States obtained from U.S. EPA: https://chemview.epa.gov/chemview/. The latest available values (2015) are given.

c *K*_ow_, octanol/water partition coefficient; NA, not available.

Considerable concern has been raised in recent years with regard to the safety of tire wear particles (TWP) and synthetic chemicals leached from rubber products.^7,8^ DPG, DTG, and TPG can be released from tire during use on roads. Animal studies showed that, following oral administration (1.5–150 μmol/kg), DPG is rapidly absorbed by the gastrointestinal tract.^9,10^ Following absorption, DPG is quickly distributed in the body, metabolized into three major and two minor metabolites (unidentified), and then excreted in urine and feces as both parent compound and metabolites.^9^ The biological half-life of DPG in rats was 9.6 h.^9^ A toxicological study reported that TWPs containing DPG was toxic to embryos of fathead minnow.^11^ The United States Environmental Protection Agency’s (EPA) ToxCast program predicated neurotoxicity and endocrine-disrupting properties of DPG.^12^ Little is known about human exposure to DPG, DTG, and TPG. One study reported the occurrence of DPG in maternal and cord serum at median concentrations of 1.7 and 0.35 ng/mL, respectively.^13^ Epidemiological and clinical studies reported association between DPG exposure and allergic contact dermatitis.^14−16^ The toxicities of DTG and TPG remain unclear. DTG was reported to exhibit reproductive and developmental toxicity in rats,^17^ whereas TPG exposure caused allergic contact dermatitis.^6^

Very few studies have investigated the occurrence of DPG, DTG, and TPG in the environment. A study measured 39 organic contaminants in stormwater samples collected from Washington State in the United States and reported the occurrence of DPG at mean concentrations of 0.54 ng/mL.^18^ DPG was found in tap water in China at concentrations in the range 0.12–0.74 ng/mL.^4^ Studies in Europe reported the occurrence of DPG (∼ 0.1 ng/mL) and DTG (∼ 0.01 ng/mL) in groundwater, surface water, and drinking water.^19−21^ Studies from Canada^22^ and Japan^23^ also reported the occurrence of DPG in surface waters.

Indoor dust has been a major source of human exposure to several environmental chemicals.^24−26^ Two earlier studies have reported the occurrence of DPG in house dust. Shin et al. collected 38 house dust samples from Northern California and found DPG in all samples at a median concentration of 3220 ng/g.^12^ DPG and DTG were frequently found in house dust samples from China (Guangzhou), Vietnam (Hanoi), Australia (Aldelaide), and the United States (Carbondale), at respective median concentrations of 5100, 305, 5030, and 11400 ng/g for DPG and 0.93, < 0.20, 2.17, and 5.81 ng/g for DTG.^27^ However, no earlier study has reported the occurrence of TPG in indoor dust. Besides, determinants of DPG levels in indoor dust (e.g., industrialization status, rural versus urban) remain unclear. Furthermore, the distribution of DPG, DTG, and TPG in various microenvironments (e.g., cars, offices, laboratories, homes, and e-waste workshops) is not known. A recent study proposed the use of DPG as a chemical marker of dust ingestion in children,^28^ which requires further validation by assessing the distribution of this chemical in dust from various countries and microenvironments.

The primary goal of this study was to comprehensively assess the occurrence and distribution pattern of DPG, DTG, and TPG in indoor dust from several countries. Dust samples were collected from 11 countries, namely, Colombia, Greece, India, Japan, Kuwait, Pakistan, Romania, Saudi Arabia, South Korea, the United States, and Vietnam during 2011–2014. Target analyte concentrations in samples from different regions (e.g., rural, urban, and suburban areas) and microenvironments (e.g., homes, cars, offices, and laboratories) were compared. Human exposure to these chemicals through dust ingestion was calculated for various age groups from 11 countries.

## 2 Materials and Methods

### 2.1 Chemicals and Reagents

The chemical structures of analytes measured in this study are shown in Table 1. Analytical standards of DPG, DTG, and TPG with purities ≥95% were purchased from Sigma-Aldrich (St. Louis, MO). Isotopically labeled internal standard of DPG (DPG-D_10_; purity ≥97%) was from Toronto Research Chemicals (Toronto, ON, Canada). LC–MS grade methanol (MeOH) and water were obtained from Fisher Scientific (Waltham, MA). Formic acid (88%) and analytical grade ammonium formate were from Sigma-Aldrich (St. Louis, MO). Individual stock solutions (1 mg/mL) of DPG, DTG, TPG, and DPG-D_10_ were prepared in MeOH. Working solutions were diluted from stock solutions using MeOH.

### 2.2 Dust Sample Collection

Details of the dust samples analyzed in this study have been described elsewhere^24^ and are given in Table S1 (Supporting Information). Briefly, a total of 332 indoor dust samples were collected from 11 countries, namely, Colombia (*n* = 44), Greece (*n* = 18), India (*n* = 28), Japan (*n* = 4), Kuwait (*n* = 29), Pakistan (*n* = 73), Romania (*n* = 20), Saudi Arabia (*n* = 34), South Korea (*n* = 41), the United States (*n* = 11), and Vietnam (*n* = 30) during 2011–2014. Dust samples were mainly collected from living rooms and bedrooms (*n* = 216), whereas in several countries, samples were also collected from microenvironments such as cars, air conditioners, offices, laboratories, and e-waste workshops (*n* = 116). Samples were collected using a vacuum cleaner or sweeping the floor. The dust samples were sieved through a 150-μm sieve and stored at 4 °C until analysis.

### 2.3 Extraction of Dust Samples

Approximately 150 mg (dry weight) of dust sample was weighed and placed into a clean 15-mL glass tube. Then, 2.5 ng of internal standard (DPG-D_10_) was spiked, followed by the addition of 5 mL of MeOH. The sample was ultrasonicated (Branson 3510 R-DTH, Branson Ultrasonics Corp., Danbury, CT) at 40 kHz for 30 min and was shaken in a reciprocal shaker (Eberbach Corp., Ann Arbor, MI) at 250 oscillations/min for 30 min. The sample was then subjected to centrifugation at 3000 rpm for 5 min (Eppendorf Centrifuge 5804, Hamburg, Germany), and the supernatant was decanted into a new glass tube. The extraction was repeated twice with 5 mL of MeOH. The extracts were combined and evaporated to dryness under a gentle nitrogen stream at 40 °C. The residue was reconstituted in 250 μL of MeOH, filtered through a 0.22 μm nylon filter (Corning, NY), transferred into a glass vial, and analyzed using high performance liquid chromatography–tandem mass spectrometry (HPLC–MS/MS).

### 2.4 Chemical Analysis

Identification and quantification of target analytes were accomplished using an ABSciex 5500+ Q-Trap mass spectrometer (Framingham, MA) coupled with an ExionLC HPLC (SCIEX, Redwood City, CA). Analytes were separated on an Ultra AQ C18 column (3 μm, 100 mm × 2.1 mm; Restek, Bellefonte, PA) connected to a BetaSil C18 guard column (5 μm, 20 mm × 2.1 mm; Thermo Fisher Scientific, Waltham, MA). The mobile phase, set at a flow rate of 0.3 mL/min, composed of 5 mM ammonium formate (A) and MeOH (B) each containing 0.1% of formic acid. The following gradient program was applied: hold at 10% B for 0.5 min, linear ramp to 90% B in 5 min, hold at 90% B for 2 min, then return to the initial condition in 0.5 min, and equilibrate for another 2 min prior to the next injection. The HPLC column and autosampler were maintained at 35 °C and 15 °C, respectively. The sample injection volume was 2 μL.

DPG, DTG, and TPG were measured in electrospray ionization (ESI) positive-ion mode. The multiple reaction monitoring (MRM) parameters including collision energy (CE), declustering potential (DP), entrance potential (EP), collision cell exit potential (CXP), and dwell time were optimized by direct infusion of a standard solution (100 ng/mL) into the mass spectrometer via a flow injection system (Table S2). The ionspray voltage was 5.5 kV; the ion source temperature was 550 °C; the pressures of curtain gas, collision gas, ion-source gas 1, and ion-source gas 2 were maintained at 20, 9, 70, and 60 psi, respectively. Data acquisition and analysis were performed using Analyst software v1.7.2 (ABSciex, Framingham, MA). Representative chromatograms of target analytes in solvent (10 ng/mL) and dust samples are shown in Figure S1.

### 2.5 Quality Assurance and Quality Control

Target analytes were quantified using an isotope dilution method. An 11-point standard calibration curve, at concentrations 0.05, 0.1, 0.2, 0.5, 1, 2, 5, 10, 20, 50, and 100 ng/mL, along with 10 ng/mL of the internal standard, was prepared in MeOH. A weighted (1/x) linear regression was applied to fit the calibration curve, which yielded regression coefficients (*R*-value) > 0.999 for all analytes (Table S3). Two procedural blanks were analyzed with each batch of 30 samples. DPG (0.46–6.33 ng/g), DTG (0.25–5.94 ng/g), and TPG (0.50–0.93 ng/g) were found in blanks of four batches, and the measured concentrations in dust were subtracted from blank values for those batches. Instrumental carryover of the target analytes was monitored by the injection of a pure solvent (i.e., MeOH) after every 10 samples. The stability of the instrumental response to analytes was monitored through the injection of a midpoint calibration standard (10 ng/mL) after every 20 samples. To estimate the limit of detection (LOD), a dust sample with target analyte concentrations of ∼1 ng/mL was measured repeatedly. The LODs were calculated as 3 times the standard deviation (SD) of the calculated concentrations, which were 0.28, 0.23, and 0.10 ng/g for DPG, DTG, and TPG, respectively. The accuracy of the method was assessed through a spike-recovery test. The recoveries of analytes spiked at 2.5, 5, 25, and 50 ng in a pooled dust (∼150 mg) were in the ranges 93.0–103%, 85.5–94.1%, and 90.9–99.9% for DPG, DTG, and TPG, respectively, with relative standard deviations of 3.8–12%, 1.6–7.9%, and 4.9–9.4%, respectively. The precision was assessed using intraday and interday variations, which were calculated as the coefficient of variation (CV%) of analyte concentrations measured in fortified dust (at 2.5, 5, 25, and 50 ng/g). The intraday variations were in the ranges 4.11–11.5%, 1.81–8.66%, and 4.86–10.4% for DPG, DTG, and TPG, respectively. The interday variations were in the ranges 1.07–2.54%, 1.91–2.95%, and 0.87–2.09% for DPG, DTG, and TPG, respectively (Table S3). A randomly selected dust sample was analyzed repeatedly over several days, which yielded CVs in the range 5.11–12.6% for all analytes.

### 2.6 Statistical Analysis

Only those analytes with detection frequencies (DFs) ≥ 50% were included in statistical analyses. Concentrations below the LOD were imputed with LOD divided by √2. The normality of the data was tested using Shapiro–Wilk test. The concentrations of DPG, DTG, and TPG between different regions and microenvironments were compared using a nonparametric test. The Spearman’s rank correlation was applied to assess the correlations between analytes. Statistical significance was set at α = 0.05. All statistical analyses were performed using R v4.1.2 (R Foundation for Statistical Computing).

## 3 Results and Discussion

### 3.1 DPG, DTG, and TPG Concentrations in House Dust

The concentrations of DPG, DTG, and TPG measured in all indoor dust samples from various countries are summarized in Table 2. The overall mean and median concentrations of ΣDPGs (sum concentrations of DPG, DTG, and TPG) in house dust were 410 and 150 ng/g, respectively. DPG was found in all dust samples (DF: 100%) with a median concentration of 140 ng/g. DTG and TPG were found with DFs of 62% and 76%, respectively, and at median concentrations of 2.3 and 0.9 ng/g, respectively. This is the first study to report the occurrence of TPG in indoor dust. One study that investigated the cause for allergic contact dermatitis reported the occurrence of TPG in gloves.^6^ DPG, DTG, and TPG are additives in rubber products that are used in tires, furniture, and shoes.^2,5^ These rubber products are the sources of these chemicals in indoor dust.

**Table 2:** Concentrations of 1,3-Diphenylguanidine (DPG), 1,3-Di-*o*-tolylguanidine (DTG), and 1,2,3-Triphenylguanidine (TPG) in House Dust (ng/g) Collected from 11 Countries during 2011–2014^a^

analytes	DF%	mean	SD	median	min	max	analytes	DF%	mean	SD	median	min	max
**Colombia** (*n* = 44)							**Romania** (*n* = 20)						
DPG	100	130	110	98	19	410	DPG	100	250	370	130	13	1700
DTG	5	8.7	9.9	<LOD	<LOD	16	DTG	80	2.8	2.9	1.8	<LOD	9.3
TPG	55	2.3	2.6	1.2	<LOD	8.3	TPG	90	1.4	1.5	1.0	<LOD	6.0
ΣDPGs^b^	100	130	110	100	19	420	ΣDPGs^b^	100	250	370	140	13	1700
**Greece** (*n* = 18)							**Saudi Arabia** (*n* = 22)						
DPG	100	1100	670	900	340	3100	DPG	100	530	350	440	140	1400
DTG	100	19	18	17	0.4	77	DTG	100	2.0	1.9	1.2	0.2	8.6
TPG	78	2.0	1.3	2.1	<LOD	4.5	TPG	100	5.0	10	2.9	0.9	50
ΣDPGs^b^	100	1100	670	940	340	3100	ΣDPGs^b^	100	540	350	440	150	1400
**India** (*n* = 28)							**South Korea** (*n* = 16)						
DPG	100	33	37	21	2.9	170	DPG	100	870	890	560	190	3700
DTG	100	4.0	2.7	3.0	0.7	10	DTG	50	8.0	19	<LOD	<LOD	55
TPG	79	0.7	0.7	0.4	<LOD	3.1	TPG	69	2.3	2.5	0.7	<LOD	8.2
ΣDPGs^b^	100	37	39	26	6.0	180	ΣDPGs^b^	100	880	900	560	190	3700
**Japan** (*n* = 4)							**USA** (*n* = 11)						
DPG	100	1300	1200	1300	130	2700	DPG	100	450	550	250	18	2000
DTG	75	15	16	13	<LOD	33	DTG	82	15	21	2.5	<LOD	52
TPG	50	3.3	1.2	<LOD	<LOD	4.2	TPG	91	1.0	0.8	0.6	<LOD	2.3
ΣDPGs^b^	100	1300	1200	1300	130	2700	ΣDPGs	100	460	560	250	18	2000
**Kuwait** (*n* = 15)							**Vietnam** (*n* = 13)						
DPG	100	250	220	150	55	670	DPG	100	1100	3000	120	17	11000
DTG	33	6.0	9.1	<LOD	<LOD	22	DTG	54	2.7	3.8	0.8	<LOD	9.9
TPG	67	1.3	1.5	0.8	<LOD	5.1	TPG	92	4.5	13	0.5	<LOD	46
ΣDPGs^b^	100	260	230	160	55	670	ΣDPGs^b^	100	1100	3000	120	18	11000
**Pakistan** (*n* = 25)							**all** (*n* = 216)						
DPG	100	42	52	32	2.1	220	DPG	100	400	910	140	2.1	11000
DTG	64	6.0	20	0.6	<LOD	82	DTG	62	7.2	13	2.3	<LOD	82
TPG	80	0.3	0.2	0.3	<LOD	0.9	TPG	76	2.1	5.4	0.9	<LOD	50
ΣDPGs^b^	100	47	55	33	2.9	220	ΣDPGs^b^	100	410	910	150	2.9	11000

a Only dust samples from living rooms or bedrooms were included in this table. Analyte concentrations were rounded to two significant digits.

b Indicate the sum concentration of DPG, DTG, and TPG.

The measured concentrations of DPG in dust from the United States (median, 250 ng/g; range, 18–2000 ng/g) in this study were 1–2 orders of magnitude lower than those reported from Northern California (median, 3220 ng/g)^12^ and Carbondale, Illinois (median, 11400 ng/g; range, 1230–25400 ng/g).^27^ The large difference in concentrations among the three different studies from the United States indicates regional difference, which can be related to vehicle traffic, sample characteristics, and time of sampling. An earlier study reported higher concentrations of DPG in watersheds from an urban area than rural area.^18^ Dust samples from our study originated from Albany (New York State), a suburban region. In fact, we also analyzed limited number of indoor dust samples collected in 2022 from New York City for comparison with those measured in Albany (Table S4). The concentrations of DPG (mean ± SD: 1700 ± 1300 ng/g in New York City vs 450 ± 550 ng/g in Albany; *p* = 0.09), DTG (33 ± 25 vs 15 ± 21 ng/g; *p* = 0.11), TPG (6.1 ± 4.3 vs 1.0 ± 0.8 ng/g; *p* = 0.04), and ΣDPGs (1700 ± 1300 vs 460 ± 560 ng/g; *p* = 0.09) in house dust collected from New York City (*n* = 5) were 2-–6-fold higher than those collected from Albany (*n* = 11), although only TPG exhibited statistical significance (Table S4 and Figure S2). Future studies with larger sample size are needed to confirm the findings. Furthermore, differences in size fractionation of dust samples analyzed between studies as well as temporal variation may affect the measured concentrations. We sieved our dust samples through a 150 μm mesh, whereas earlier studies used a greater mesh size.

DPG concentration measured in dust from Vietnam (median, 120 ng/g; range, 17–11000 ng/g) in our study was in the same range as that reported in an earlier study (median, 305 ng/g; range, 53.6–2610 ng/g).^27^ The concentrations of DTG measured in dust from the United States (median: 2.5 ng/g in our study vs 5.81 ng/g in an earlier study) and Vietnam (median: 0.8 ng/g in our study vs < LOD in an earlier study) in our study were similar to those reported earlier.^27^

**Figure 1:**
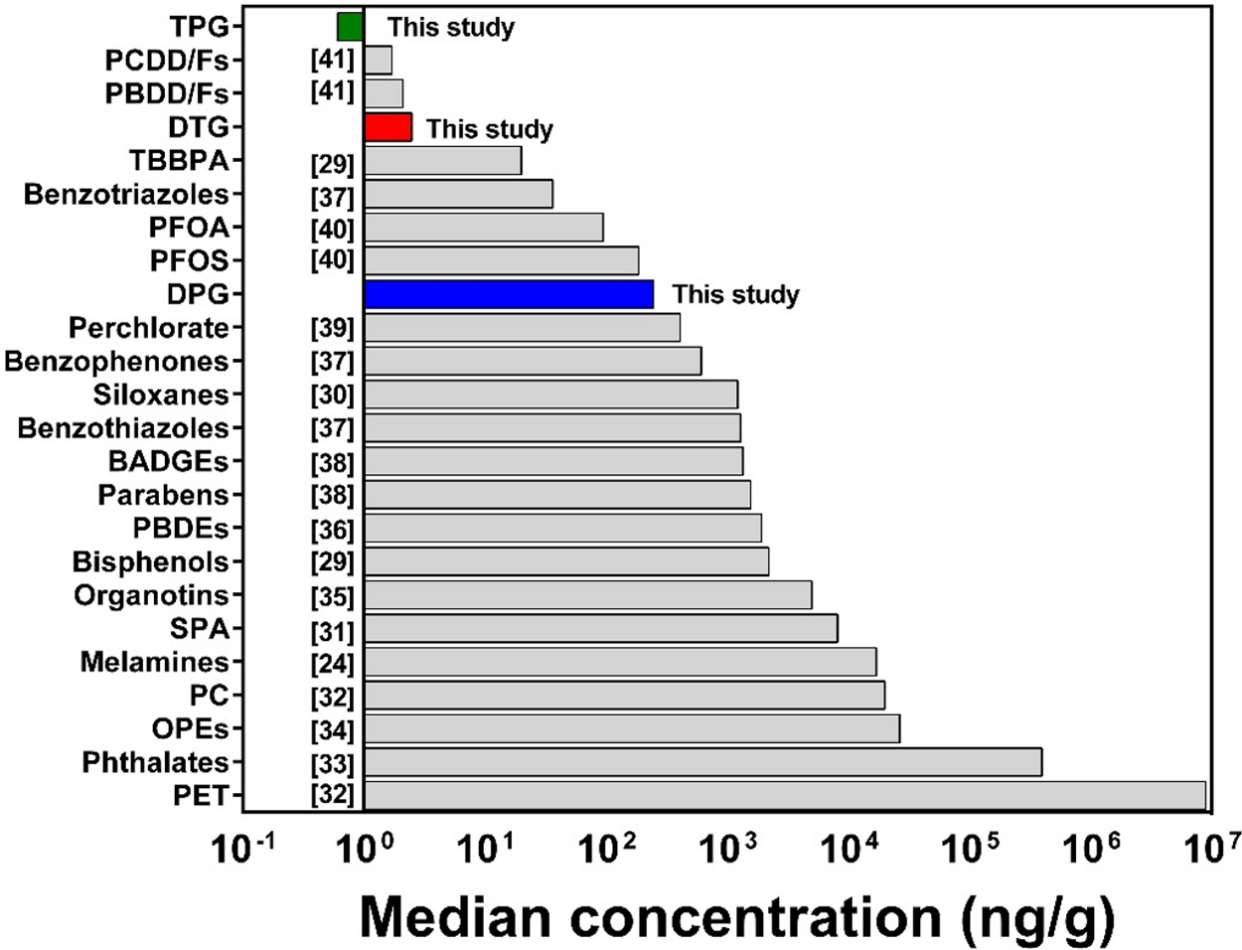
Comparison of median concentrations of 1,3-diphenylguanidine (DPG), 1,3-di-*o*-tolylguanidine (DTG), and 1,2,3-triphenylguanidine (TPG) measured in house dust from Albany, New York State, the United States, in this study against other chemicals reported previously. PET, polyethylene terephthalate-based microplastics; OPEs, organophosphate esters; PC, polycarbonate-based microplastics; SPA, synthetic phenolic antioxidants; PBDEs, polybrominated diphenyl ethers; BADGEs, bisphenol A diglycidyl ethers; PFOS, perfluorooctanesulfonate; PFOA, perfluorooctanoic acid; TBBPA, tetrabromobisphenol A; PBDD/Fs, polybrominated dibenzo-*p*-dioxins/furans; PCDD/Fs, polychlorinated dibenzo-*p*-dioxins/furans.

The measured concentrations of DPG, DTG, and TPG were compared with other environmental chemicals determined in the same house dust collected from Albany, New York in 2014 (Figure 1). DPG ranked 16^th^ highest among the 22 chemical classes analyzed in the same set of dust samples.^24,29−41^ The median concentration of DPG (250 ng/g) was close to those of perchlorate (410 ng/g)^39^ and perfluorooctanesulfonate (PFOS; 185 ng/g)^40^ but up to 2 orders of magnitude higher than those of polychlorinated dibenzo-*p*-dioxins/furans (PCDD/Fs; median: 1.7 ng/g),^41^ polybrominated dibenzo-*p*-dioxins/furans (PBDD/Fs; 2.1 ng/g),^41^ tetrabromobisphenol A (TBBPA; 20 ng/g),^29^ benzotriazoles (36.2 ng/g),^37^ and perfluorooctanoic acid (PFOA; 94.5 ng/g).^40^ In particular, benzotriazoles and benzothiazoles are also used as rubber additives.^37^ The median concentration of ΣDPG was 7-fold higher than that of benzotriazoles but 5-fold lower than that of benzothiazoles (Figure 1). The toxic potencies of DPG relative to other chemicals is not well-known. Nevertheless, our results highlight the significance of DPG in the indoor environment.

### 3.2 Correlations, Profiles, and Geographical Patterns

**Figure 2:**
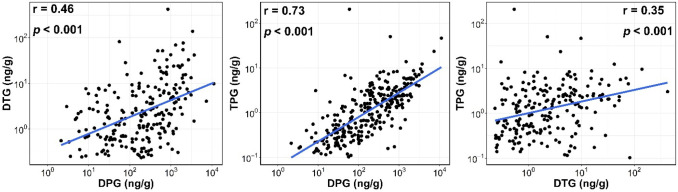
Scatterplots and Spearman’s rank correlations (a) between 1,3-diphenylguanidine (DPG) and 1,3-di-*o*-tolylguanidine (DTG), (b) between DPG and 1,2,3-triphenylguanidine (TPG), and (c) between DTG and TPG measured in indoor dust samples collected from 11 countries. Analyte concentrations were log_10_-transformed.

**Figure 3:**
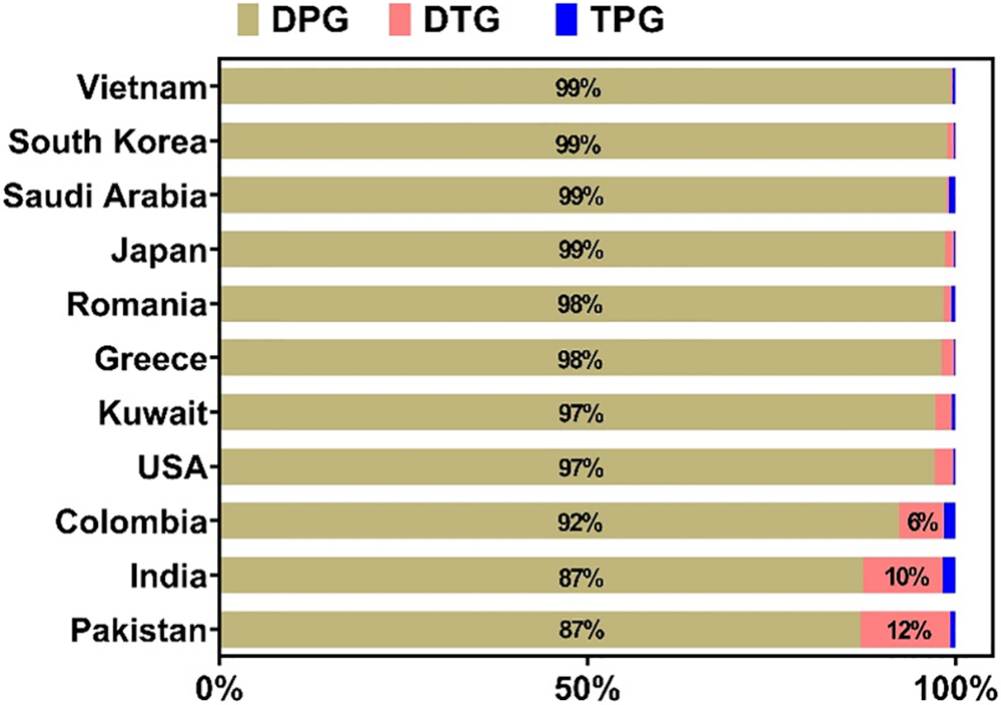
Stack distribution of 1,3-diphenylguanidine (DPG), 1,3-di-*o*-tolylguanidine (DTG), and 1,2,3-triphenylguanidine (TPG) in house dust (living rooms or bedrooms) collected from 11 countries.

DPG was significantly correlated with DTG (*r* = 0.46, *p* < 0.001) and TPG (*r* = 0.73, *p* < 0.001) (Figure 2), suggesting common sources/origins of the three analytes in house dust. DPG accounted for >97% of the sum concentrations of the three compounds in house dust samples from Vietnam, South Korea, Saudi Arabia, Japan, Romania, Greece, Kuwait, and the United States. However, in samples from India and Pakistan, DPG accounted for 87% of the total concentrations, while DTG contributed 10–12% of the total concentrations (Figure 3). A greater proportion of DTG implies that this compound is also an additive used in rubber products in India and Pakistan. In dust samples collected from the United States, the distribution profiles of DPG, DTG, and TPG corresponded to their consumption volume (Table 1).

**Figure 4:**
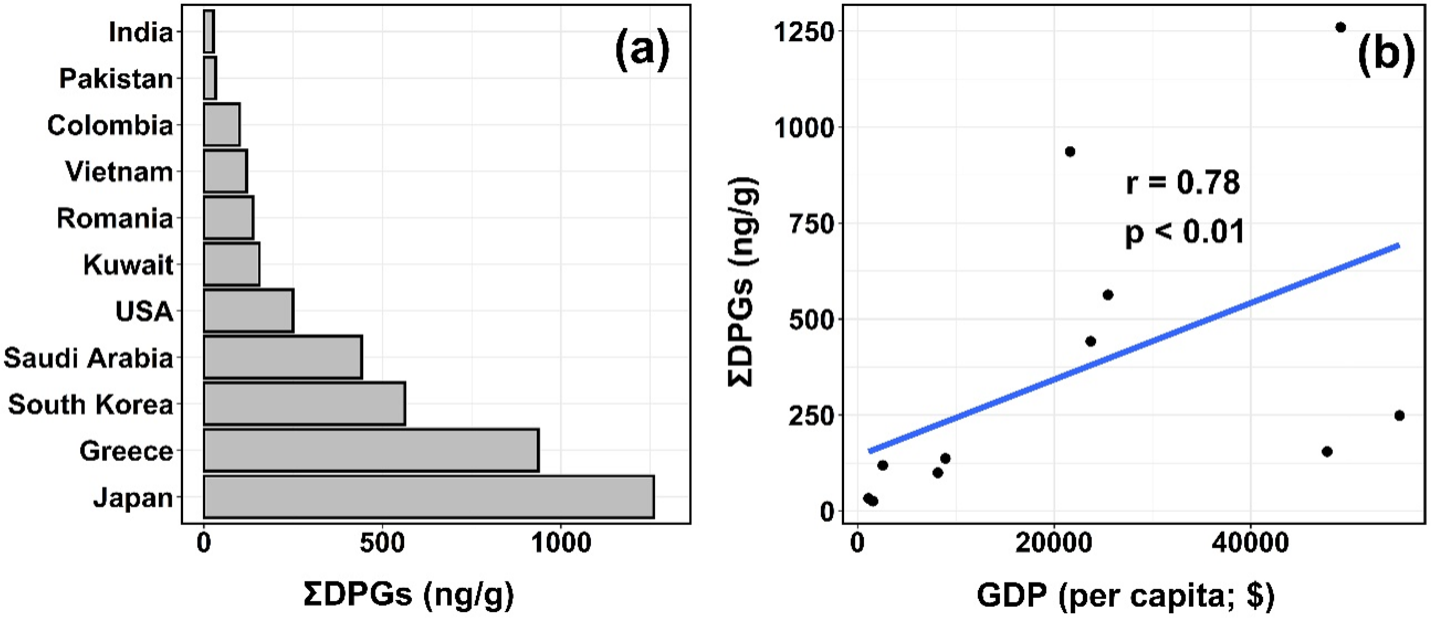
(a) Median concentrations (ng/g) of DPG and its analogues (ΣDPGs; sum of DPG, DTG, and TPG) in house dust collected from 11 countries. (b) Scatterplots and Spearman’s correlation between median concentrations ΣDPGs and gross domestic product (GDP) per capita of the 11 countries in the year of sample collection. Samples collected in the United States indicate those collected from Albany, New York State in 2014.

DPG, DTG, and TPG concentrations in indoor dust samples exhibited a characteristic geographical pattern. DTG and TPG were found in all house dust samples collected from Saudi Arabia, but they were found in only 5% and 55%, respectively, of the house dust samples from Colombia. As mentioned above, DTG contributed to a higher proportion of the total concentrations in dust from India and Pakistan. These results suggest different usage patterns of these compounds among various countries. Furthermore, ΣDPG concentrations measured in dust samples from more industrialized countries such as Japan (median: 1300 ng/g), Greece (940 ng/g), South Korea (560 ng/g), Saudi Arabia (440 ng/g), and the United States (250 ng/g) were higher than those in dust from less industrialized countries such as Kuwait (160 ng/g), Romania (140 ng/g), Vietnam (120 ng/g), Colombia (100 ng/g), Pakistan (33 ng/g), and India (26 ng/g) (Figure 4a). This may be related to the consumption of rubber products (e.g., tire) per capita. For example, the estimated emission of TWPs per capita in the United States was 4.7 kg/year, which was 20-fold higher than that in India (0.23 kg/year).^42^ This generally agrees with the measured DPG concentrations in dust from the two countries (median: 250 and 21 ng/g for the United States and India, respectively). The median concentrations of ΣDPG exhibited a strong and significant positive correlation with the gross domestic product (GDP) per capita of the countries studied (*r* = 0.78, *p* < 0.01) (Figure 4b). The spatial distribution pattern of ΣDPG in house dust is generally consistent with those reported for TBBPA,^29^ siloxanes,^30^ synthetic phenolic antioxidants (SPAs),^31^ melamine and its derivatives,^24^ and microplastics,^32^ indicating greater exposure to indoor environmental chemicals in highly industrialized countries.

### 3.3 DPG, DTG, and TPG in Dust from Microenvironments

The concentrations of DPG, DTG, and TPG in indoor dust varied widely among various microenvironments (Table S5 and Figures S3–S7). In South Korea, the concentrations (mean ± SD) of DPG in dust from offices, laboratories, and homes were 1400 ± 960, 1300 ± 530, and 870 ± 890 ng/g (*p* = 0.03), respectively. The concentrations of DTG (mean ± SD: 48 ± 110, 7.9 ± 6.4, and 8.0 ± 19 ng/g, respectively; *p* < 0.0001), TPG (mean ± SD: 3.8 ± 2.8, 3.5 ± 2.2, and 2.3 ± 2.5 ng/g, respectively; *p* = 0.003), and ΣDPGs (mean ± SD: 1500 ± 970, 1400 ± 540, and 880 ± 900 ng/g, respectively; *p* = 0.03) in dust from the three microenvironments in South Korea were significantly different, with the concentrations measured in offices and laboratories higher than those from homes (Figure S3). This suggests that furniture and equipment in offices and laboratories may contribute to higher levels of DPG, DTG, and TPG. Similarly, our earlier study found higher levels of SPAs in office dust than in house dust from South Korea.^31^ A recent study reported positive correlation between concentrations of DPG and SPAs (e.g., 3,5-di-*tert*-butyl-4-hydroxybenzaldehyde) measured in house dust.^27^

In Kuwait, the concentrations of DPG (mean ± SD: 1000 ± 2000 ng/g in cars vs 250 ± 220 ng/g in homes; *p* = 0.08), TPG (mean ± SD: 4.6 ± 6.0 vs 0.9 ± 1.3 ng/g; *p* = 0.0004), and ΣDPGs (mean ± SD: 1100 ± 2000 vs 260 ± 230 ng/g; *p* = 0.10) in dust collected from cars were 4-–5-fold higher than in those collected from homes, although only TPG exhibited statistical significance (Figure S4). This could be explained by sources arising from tire wear particles in cars.

Although ΣDPG concentrations were higher in dust from New York City than those from Albany, no significant difference was found in dust from rural and urban areas of Pakistan (Figure S5). Besides, the concentrations of DPG, DTG, and TPG in house dust were not significantly different from those of offices and cars in Pakistan. This may suggest sources other than traffic contribute to DPG in the indoor environment. Although DPG accounted for >96% of the total concentrations in all microenvironments from other countries, DTG and TPG accounted for 17% and 16%, respectively, in dust from urban homes and offices in Pakistan (Figure S5b). These results indicate a different profile of vulcanization additives used in Pakistan.

The concentrations of DPG (mean ± SD: 530 ± 350 and 180 ± 55 ng/g in dust from homes and cars, respectively; *p* < 0.001) and ΣDPGs (mean ± SD: 540 ± 350 and 180 ± 57 ng/g, respectively; *p* < 0.001) measured in dust from homes were significantly higher than those in car dust collected from Saudi Arabia (Figure S6). These results indicate that, apart from vehicle traffic, other indoor sources contribute to the occurrence of DPG in dust.

In Vietnam, the concentrations of DPG (mean ± SD: 360 ± 230 ng/g in e-waste workshops vs 97 ± 110 ng/g in homes; *p* = 0.02), TPG (mean ± SD: 2.4 ± 1.5 vs 0.4 ± 0.2 ng/g; *p* = 0.01), and ΣDPGs (mean ± SD: 360 ± 230 vs 98 ± 110 ng/g; *p* = 0.02) in dust collected from e-waste dismantling workshops were significantly higher than those from homes (Table S5 and Figure S7). In particular, the highest DPG concentration (of 11000 ng/g; Table S5) measured in this study was in dust from a room in an e-waste dismantling site in Vietnam. These results suggest usage of DPG and TPG in electric/electronic products and e-waste sites are significant sources of these chemicals in the surrounding environment.^5^

### 3.4 Exposure Assessment

We estimated exposure to DPG, DTG, and TPG through dust ingestion for various age groups using the following equation:
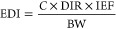
1where EDI is the estimated daily intake (ng/kg body weight (BW)/day) and *C* is the median concentration of the analytes measured in house dust (ng/g). Only samples collected from living rooms or bedrooms were included in this calculation. DIR is the daily dust ingestion rate, which was reported to be 20, 100, 50, 50, and 50 mg/day for infants (<1 y), toddlers (1–5 y), children (6–11 y), teenagers (12–19 y), and adults (≥20 y), respectively.^43^ BW is the body weight (kg), which was reported to be 5, 19, 29, 53, and 63 kg, respectively, for infants, toddlers, children, teenagers, and adults from Asian countries and 7, 15, 32, 64, and 80 kg, respectively, for those from western countries.^24^ IEF refers to the indoor exposure fraction (fraction of the time spent indoor), which was reported to be 88%, 79%, 79%, 88%, and 88% for infants, toddlers, children, teenagers, and adults, respectively.^27^ We assumed an absorption rate of 100% for DPG, DTG, and TPG through dust ingestion.^9^ The detailed parameters used for EDI calculation are summarized in Table S6.

The median EDIs of DPG for infants, toddlers, children, teenagers, and adults were in the ranges 0.07–4.40, 0.09–5.20, 0.03–1.70, 0.02–1.04, and 0.01–0.87 ng/kg BW/day, respectively (Table 3). Similar to other environmental chemicals (e.g., bisphenols,^43^ phthalatess^33^ and melamine^24^), the highest exposure to DPG through dust ingestion was among toddlers and infants, which is due to their high dust ingestion rate and low body weight. This is of critical importance because infants and toddlers are generally more sensitive and vulnerable to chemical exposure.^44^ The highest exposure dose to DPG among toddlers was found for those from Japan (median: 5.20 ng/kg BW/day), which was 2 orders of magnitude higher than that calculated for Indian toddlers (0.09 ng/kg BW/day). The EDIs of DTG and TPG for all age groups from all countries through dust ingestion were ≤0.09 and ≤0.01 ng/kg BW/day, respectively (Table 3).

**Table 3:** Median Estimated Daily Intakes (EDI; ng/kg-bw/day) of 1,3-Diphenylguanidine (DPG), 1,3-Di-*o*-tolylguanidine (DTG), and 1,2,3-Triphenylguanidine (TPG) through Indoor Dust Ingestion for Infants (< 1 y), Toddlers (1–5 y), Children (6–11 y), Teenagers (12–19 y), and Adults (≥ 20 y) from 11 Countries Collected during 2011–2014^a^

	EDI (ng/kg/day)						EDI (ng/kg/day)				
	infants	toddlers	children	teenagers	adults		infants	toddlers	children	teenagers	adults
**Colombia**						**Romania**					
DPG	0.25	0.52	0.12	0.07	0.05	DPG	0.33	0.68	0.16	0.09	0.07
DTG	NC^d^	NC^d^	NC^d^	NC^d^	NC^d^	DTG	0.004	0.01	0.002	0.001	0.001
TPG	0.003	0.01	0.001	0.001	0.001	TPG	0.003	0.005	0.001	0.001	0.001
ΣDPGs^b^	0.25	0.53	0.12	0.07	0.05	ΣDPGs^b^	0.34	0.72	0.17	0.09	0.08
**Greece**						**Saudi Arabia**					
DPG	2.27	4.75	1.11	0.62	0.50	DPG	1.53	1.81	0.59	0.36	0.30
DTG	0.04	0.09	0.02	0.01	0.01	DTG	0.004	0.01	0.002	0.001	0.001
TPG	0.01	0.01	0.003	0.001	0.001	TPG	0.01	0.01	0.004	0.002	0.002
ΣDPGs^b^	2.35	4.93	1.16	0.64	0.51	ΣDPGs^b^	1.56	1.84	0.60	0.37	0.31
**India**						**South Korea**					
DPG	0.07	0.09	0.03	0.02	0.01	DPG	1.98	2.34	0.77	0.47	0.39
DTG	0.01	0.01	0.004	0.002	0.002	DTG	NC^d^	NC^d^	NC^d^	NC^d^	NC^d^
TPG	0.001	0.001	0.0005	0.0003	0.0003	TPG	0.005	0.01	0.002	0.001	0.001
ΣDPGs^b^	0.09	0.11	0.04	0.02	0.02	ΣDPGs^b^	1.98	2.34	0.77	0.47	0.39
**Japan**						**USA**^c^					
DPG	4.40	5.20	1.70	1.04	0.87	DPG	0.63	1.32	0.31	0.17	0.14
DTG	0.05	0.05	0.02	0.01	0.01	DTG	0.01	0.01	0.003	0.002	0.001
TPG	NC^d^	NC^d^	NC^d^	NC^d^	NC^d^	TPG	0.002	0.003	0.001	0.0004	0.0003
ΣDPGs^b^	4.44	5.24	1.72	1.05	0.88	ΣDPGs^b^	0.63	1.32	0.31	0.17	0.14
**Kuwait**						**Vietnam**					
DPG	0.54	0.64	0.21	0.13	0.11	DPG	0.42	0.49	0.16	0.10	0.08
DTG	NC^d^	NC^d^	NC^d^	NC^d^	NC^d^	DTG	0.003	0.003	0.001	0.001	0.001
TPG	0.003	0.003	0.001	0.001	0.001	TPG	0.002	0.002	0.001	0.0004	0.0004
ΣDPGs^b^	0.55	0.64	0.21	0.13	0.11	ΣDPGs^b^	0.42	0.49	0.16	0.10	0.08
**Pakistan**						**all**^c^					
DPG	0.11	0.13	0.04	0.03	0.02	DPG	0.50	0.59	0.19	0.12	0.10
DTG	0.002	0.003	0.001	0.001	0.0004	DTG	0.002	0.003	0.001	0.001	0.0005
TPG	0.001	0.001	0.0004	0.0003	0.0002	TPG	0.002	0.002	0.001	0.0004	0.0003
ΣDPGs^b^	0.12	0.14	0.05	0.03	0.02	ΣDPGs^b^	0.52	0.61	0.20	0.12	0.10

a Only dust samples collected from living rooms and bedrooms were included in this analysis.

b Calculated from the total concentrations of DPG, DTG, and TPG.

c Samples from the United States indicate those collected from Albany, New York State (*n* = 11) in 2014.

d NC, not calculated.

The median EDIs of a wide range of environmental chemicals through dust ingestion were calculated for different age groups in the United States (Table 4). The EDI of DPGs was comparable to those of PFOS and perchlorate but was up to 2 orders of magnitude higher than that of PCDD/Fs, PBDD/Fs, TBBPA, benzotriazoles, and PFOA. The toxicity reference doses (RfDs) of DPG, DTG, and TPG are not currently available. In comparison to RfD available for diphenylamine (25000 ng/kg BW/day),^45^ which is structurally similar to DPG, the median EDIs of DPG, DTG, and TPG were at least 4 orders of magnitude lower. These results suggest that dust ingestion alone is a minor contributor to health risks of DPG, DTG, and TPG, although establishment of evidence-based human toxicity reference values is essential to assess risks of this class of chemicals.

**Table 4:** Comparison of Estimated Daily Intake (EDI) Values Calculated for DPGs against Other Environmental Chemicals Measured in House Dust Samples Collected from Albany, New York State^a^

	EDI (ng/kg BW/day)					
compounds	infants	toddlers	children	teenagers	adults	references
PCDD/Fs	0.004	0.009	0.002	0.001	0.001	(41)
PBDD/Fs	0.005	0.011	0.003	0.001	0.001	(41)
TBBPA	0.05	0.11	0.02	0.01	0.01	(29)
benzotriazoles	0.09	0.19	0.04	0.02	0.02	(37)
PFOA	0.24	0.50	0.12	0.06	0.05	(40)
PFOS	0.47	0.97	0.23	0.13	0.10	(40)
DPGs	0.63	1.31	0.31	0.17	0.14	this study
perchlorate	1.03	2.16	0.51	0.28	0.23	(39)
benzophenones	1.54	3.22	0.76	0.42	0.34	(37)
siloxanes	3.07	6.43	1.51	0.84	0.67	(30)
benzothiazoles	3.24	6.79	1.59	0.89	0.71	(37)
BADGEs	3.39	7.11	1.67	0.93	0.74	(38)
parabens	3.92	8.22	1.93	1.07	0.86	(38)
PBDEs	4.80	10.1	2.36	1.31	1.05	(36)
bisphenols	5.53	11.6	2.72	1.51	1.21	(29)
organotins	12.6	26.3	6.17	3.44	2.75	(35)
SPA	20.5	43.0	10.1	5.62	4.49	(31)
melamines	42.7	89.5	21.0	11.7	9.35	(24)
PC	50.3	105	24.7	13.8	11.0	(32)
OPEs	66.6	140	32.7	18.2	14.6	(34)
phthalates	996	2090	489	272	218	(33)
PET	22400	46900	11000	6120	4900	(32)

a Abbreviations: PET, polyethylene terephthalate-based microplastics; OPEs, organophosphate esters; PC, polycarbonate-based microplastics; SPA, synthetic phenolic antioxidants; PBDEs, polybrominated diphenyl ethers; BADGEs, bisphenol A diglycidyl ethers; PFOS, perfluorooctanesulfonate; PFOA, perfluorooctanoic acid; TBBPA, tetrabromobisphenol A; PBDD/Fs, polybrominated dibenzo-*p*-dioxins/furans; PCDD/Fs, polychlorinated dibenzo-*p*-dioxins/furans.

It should be noted that several uncertainties exist in our exposure assessment of DPG, DTG, and TPG through dust ingestion. For example, apart from age, the rate of dust ingestion can be affected by factors such as personal habits and living environment. Furthermore, humans can be exposed to DPG through dermal, inhalation, and dietary pathways.^4,19,20^ DPG concentrations measured in dust from certain microenvironments (e.g., offices, cars, and e-waste workshops) were higher than those in dust from living rooms or bedrooms (Figures S3–S7), which may augment exposures. Finally, it should be noted that the health effects of DPG following chronic, long-term exposure remain unknown.

This is the first comprehensive assessment of DPG, DTG, and TPG in indoor dust from 11 countries and provides critical baseline information on their occurrence in the indoor environment. Nevertheless, the sample size was small for several microenvironments, and therefore, the comparison is limited in statistical power.

## Supporting Information Available

Tables of information on the indoor dust samples analyzed in this study, target analytes, isotopically labeled internal standard, and their optimized MRM parameters,method validation parameters, analyte concentrations in indoor dust from New York City, comparison of analyte concentrations in samples from different microenvironments, andparameters for exposure assessment and figures of representative chromatograms of all analytes and comparisons of the concentrations (PDF)
