# Supplementary material for: Occurrence of 1,3-Diphenylguanidine, 1,3-Di-o-tolylguanidine, and 1,2,3-Triphenylguanidine in Indoor Dust from 11 Countries: Implications for Human Exposure
Source: Environ Sci Technol. 2023 Apr 3;57(15):6129–38. doi: 10.1021/acs.est.3c00836 (PMC10116588; doi:10.1021/acs.est.3c00836)
Supplement: Supplementary file 1 — es3c00836_si_001.pdf [file es3c00836_si_001.pdf]

Supporting Information

Occurrence of 1,3-Diphenylguanidine, 1,3-Di-*o*-tolylguanidine, and 1,2,3-Triphenylguanidine in Indoor Dust from 11 Countries: Implications for Human Exposure

Zhong-Min Li[a,b] and Kurunthachalam Kannan*[a,b]

[a]Department of Pediatrics, New York University Grossman School of Medicine, New York, NY 10016, United States

[b]Department of Environmental Medicine, New York University Grossman School of Medicine, New York, NY 10016, United States

***Corresponding Author**

Kurunthachalam Kannan

Address: MSB 6-698, 550 First Avenue, New York, NY 10016, United States

Tel.: (212)-263-1546

E-mail: kurunthachalam.kannan@health.ny.gov

Number of pages: 19

Number of figures: 7

Number of tables: 6

Table of contents

Table S1 Details of dust samples investigated in this study S4

Table S2 Target analytes, isotopically labeled internal standard, and their optimized MRM parameters. Two transitions were optimized for each analyte, one for quantification (q) and the other for confirmation (c). One transition was optimized for the internal standard. MRM parameters include precursor ion (Q1), product ion (Q3), declustering potential (DP), entrance potential (EP), collision energy (CE), collision cell exit potential (CXP), and dwell time S5

Table S3 Method validation parameters for the determination of 1,3-diphenylguanidine (DPG), 1,3-di-*o*-tolylguanidine (DTG), and 1,2,3-triphenylguanidine (TPG) in dust S6

Table S4 Concentrations of 1,3-diphenylguanidine (DPG), 1,3-di-*o*-tolylguanidine (DTG), and 1,2,3-triphenylguanidine (TPG) in 5 house dust samples collected from New York City, the United States in 2022 S7

Table S5 Concentrations of 1,3-diphenylguanidine (DPG), 1,3-di-*o*-tolylguanidine (DTG), and 1,2,3-triphenylguanidine (TPG) in dust collected from various microenvironment in several countries S8

Table S6 Parameters used for the estimation of human exposure to 1,3-diphenylguanidine (DPG) and derivatives through ingestion of indoor dust S11

Figure S1 Representative chromatograms of 1,3-diphenylguanidine (DPG), 1,3-di-*o*-tolylguanidine (DTG), and 1,2,3-triphenylguanidine (TPG) in standard solution (10 ng/mL) and in dust samples. Injection volume was 2 µL S12

Figure S2 (a) Comparison of the concentrations of 1,3-diphenylguanidine (DPG), 1,3-di-*o*-tolylguanidine (DTG), 1,2,3-triphenylguanidine (TPG), and the sum of DPG and analogues (ΣDPGs) in house dust collected from Albany (n = 11) and New York City (n = 5), United States. The concentrations were compared using nonparametric test. # $p < 0.1$; * $p < 0.05$; p-values > 0.1 are not shown. (b) Stack distribution of DPG, DTG and TPG in house dust collected from Albany and New York City S13

Figure S3 (a) Comparison of the concentrations of 1,3-diphenylguanidine (DPG), 1,3-di-*o*-tolylguanidine (DTG), 1,2,3-triphenylguanidine (TPG), and the sum of DPG and analogues (ΣDPGs) in dust collected from houses, laboratories, and offices of South Korea. The analyte concentrations among different microenvironments were compared using nonparametric test. # $p < 0.1$; * $p < 0.05$; ** $p < 0.01$; p-values > 0.1 are not shown. (b) Stack distribution of DPG, DTG and TPG in dust collected from houses, offices and laboratories in South Korea S14

Figure S4 (a) Comparison of concentrations of 1,3-diphenylguanidine (DPG), 1,3-di-*o*-tolylguanidine (DTG), 1,2,3-triphenylguanidine (TPG), and the sum of DPG and analogues (ΣDPGs) collected from car and house in Kuwait. DTG was not included due to its low detection frequency (DF; 33%) in house dust. Nonparametric test was use for the comparison. # $p < 0.1$; ** $p < 0.01$; p-values > 0.1 are not shown. (b) Stack distribution of DPG, DTG and TPG in dust collected from cars and houses of Kuwait S15

Figure S5 (a) Comparison of the concentrations of 1,3-diphenylguanidine (DPG), 1,3-di-*o*-tolylguanidine (DTG), 1,2,3-triphenylguanidine (TPG), and the sum of DPG and analogues (ΣDPGs) in dust from car, rural house, urban house and office of Pakistan. # $p < 0.1$; p-values >

0.1 are not shown. (b) Stack distribution of DPG, DTG and TPG in dust collected from cars, urban houses, rural houses, and offices of Pakistan S16

Figure S6 (a) Comparison of concentrations of 1,3-diphenylguanidine (DPG), 1,3-di-*o*-tolylguanidine (DTG), 1,2,3-triphenylguanidine (TPG), and the sum of DPG and analogues (ΣDPGs) collected from house, air conditioner, and car in Saudi Arabia. Nonparametric test was use for the comparison. ** $p < 0.01$; p-values > 0.1 are not shown. (b) Stack distribution of DPG, DTG and TPG in dust collected from houses, air conditioners and cars of Saudi Arabia S17

Figure S7 (a) Comparison of concentrations of 1,3-diphenylguanidine (DPG), 1,3-di-*o*-tolylguanidine (DTG), 1,2,3-triphenylguanidine (TPG), and the sum of DPG and analogues (ΣDPGs) collected from E-waste workshops and homes of Vietnam. Nonparametric test was use for the comparison. # $p < 0.1$; * $p < 0.05$; p-values > 0.1 are not shown. (b) Stack distribution of DPG, DTG and TPG in dust collected from houses (living rooms), e-waste workshops, e-waste living rooms, offices, and supermarkets of Vietnam S18

References S19

Table S1 Details of dust samples investigated in this study.

Country	Area	Location	Year	Number
Colombia	Cartagena (urban area)	Home	2014	44
Greece	Athens, Erateini, Komotini	Home	2014	18
India	Patna (urban area)	Home	2014	28
Japan	Kumaooto, Nagasaki, Fukuoka, Saitama, Saga	Home	2012	4
Kuwait	Kuwait	Car	2013	14
	Kuwait (urban area)	Home		15
Pakistan	Faisalabad	Car	2011–2012	6
	Faisalabad (rural area)	Rural home		13
	Faisalabad (urban area)	Urban home		12
	Faisalabad (urban area)	Office		42
Romania	Iasi (urban area)	Home	2012	20
Saudi Arabia	Jeddah (urban area)	Home	2013	22
		Air conditioner		3
		Car		9
South Korea	Ansan, Anyang (urban area)	Laboratory	2012	11
		Office		14
		Home		16
U.S.A.	New York City (urban area)	Home	2022	5
	Albany (urban area)	Home	2014	11
Vietnam	Hanoi, Hatinh, Hungyen, Thaibinh	Electronic workshop	2014	7
		Supermarket		4
		Electronic living room		8
		Office		4
		Living room		5
		Kitchen		2

Table S2 Target analytes, isotopically labeled internal standard, and their optimized MRM parameters. Two transitions were optimized for each analyte, one for quantification (q) and the other for confirmation (c). One transition was optimized for the internal standard. MRM parameters include precursor ion (Q1), product ion (Q3), declustering potential (DP), entrance potential (EP), collision energy (CE), collision cell exit potential (CXP), and dwell time.

Analytes	Abbr.	Q1 (*m/z*)	Q3 (*m/z*)	DP (V)	EP (V)	CE (V)	CXP (V)	Dwell time (msec)
**Target analytes**								
1,3-Diphenylguanidine	DPG	212	119 (q)	100	31	10	10	80
			77 (c)	80	51	10	12	80
1,3-Di-*o*-tolylguanidine	DTG	240	133 (q)	98	32	10	9	80
			108 (c)	11	31	10	30	80
1,2,3-Triphenylguanidine	TPG	288	195 (q)	100	33	10	16	80
			92 (c)	100	45	10	12	80
**Internal standard**								
1,3-Diphenylguanidine-D_{10}	DPG-D_{10}	222	124	100	31	10	15	80

Table S3 Method validation parameters for the determination of 1,3-diphenylguanidine (DPG), 1,3-di-*o*-tolylguanidine (DTG), and 1,2,3-triphenylguanidine (TPG) in dust.

	DPG	DTG	TPG
R	0.9997	0.9996	0.9993
LOD (ng/g)	0.28	0.23	0.10
Spike-recovery (%), *n* = 3			
2.5 ng	103 ± 6	85.5 ± 5.3	93.7 ± 8.6
5 ng	100 ± 12	88.8 ± 1.6	99.9 ± 4.9
25 ng	102 ± 8	94.1 ± 5.7	99.5 ± 5.6
50 ng	93.0 ± 3.8	91.7 ± 7.9	90.9 ± 9.4
Intra-day variation (%), *n* = 3			
2.5 ng	5.59	6.23	9.20
5 ng	11.5	1.81	4.86
25 ng	7.92	6.07	5.66
50 ng	4.11	8.66	10.4
Inter-day variation (%), *n* = 3			
2.5 ng	1.07	2.95	2.09
5 ng	1.72	1.91	1.00
25 ng	1.45	2.15	0.87
50 ng	2.54	1.97	2.07

Table S4 Concentrations of 1,3-diphenylguanidine (DPG), 1,3-di-*o*-tolylguanidine (DTG), and 1,2,3-triphenylguanidine (TPG) in 5 house dust samples collected from New York City, the United States in 2022.

	DPG	DTG	TPG	ΣDPG
DF%	100	100	100	100
Min (ng/g)	79	2.1	0.3	110
Median (ng/g)	1500	29	6.0	1500
Max (ng/g)	3700	70	12	3800
Mean (ng/g)	1700	33	6.1	1700
SD	1300	25	4.3	1300

Table S5 Concentrations of 1,3-diphenylguanidine (DPG), 1,3-di-*o*-tolylguanidine (DTG), and 1,2,3-triphenylguanidine (TPG) in dust collected from various microenvironment in several countries.

			DPG	DTG	TPG	ΣDPG
Pakistan	Car (*n* = 6)	DF%	100	33	100	100
		Min (ng/g)	29	< LOD	0.2	30
		Median (ng/g)	31	< LOD	0.3	32
		Max (ng/g)	97	1.8	0.4	97
		Mean (ng/g)	44	1.2	0.3	44
		SD	27	0.9	0.1	27
	Rural house (*n* = 13)	DF%	100	62	69	100
		Min (ng/g)	3.4	< LOD	< LOD	4.0
		Median (ng/g)	26	0.4	0.2	27
		Max (ng/g)	220	82	0.4	220
		Mean (ng/g)	53	11	0.3	60
		SD	70	29	0.1	74
	Urban house (*n* = 12)	DF%	100	67	92	100
		Min (ng/g)	2.1	< LOD	< LOD	2.9
		Median (ng/g)	32	0.6	0.3	34
		Max (ng/g)	69	2.4	0.9	70
		Mean (ng/g)	31	1.0	0.4	32
		SD	18	0.7	0.2	18
	Office (*n* = 42)	DF%	100	69	88	100
		Min (ng/g)	0.6	< LOD	< LOD	0.6
		Median (ng/g)	26	0.4	0.4	27
		Max (ng/g)	160	2.7	210	270
		Mean (ng/g)	32	0.7	6.1	38
		SD	30	0.5	34	47
South Korea	House (*n* = 16)	DF%	100	50	69	100
		Min (ng/g)	190	< LOD	< LOD	190
		Median (ng/g)	560	< LOD	0.7	560
		Max (ng/g)	3700	55	8.2	3700
		Mean (ng/g)	870	8.0	2.3	880
		SD	890	19	2.5	900
	Laboratory (*n* = 11)	DF%	100	100	100	100
		Min (ng/g)	490	2.1	1.3	500
		Median (ng/g)	1300	6.1	2.6	1300
		Max (ng/g)	2300	24	8.7	2300
		Mean (ng/g)	1300	7.9	3.5	1400
		SD	530	6.4	2.2	540
	Office (*n* = 14)	DF%	100	100	100	100
		Min (ng/g)	270	0.3	0.6	270

		Median (ng/g)	1200	7.1	2.7	1300
		Max (ng/g)	3400	430	9.5	3500
		Mean (ng/g)	1400	48	3.8	1500
		SD	960	110	2.8	970
Kuwait	Car (*n* = 14)	DF%	100	64	100	100
		Min (ng/g)	65	< LOD	0.5	66
		Median (ng/g)	410	4.3	2.5	420
		Max (ng/g)	7300	17	24	7300
		Mean (ng/g)	1000	6.3	4.6	1100
		SD	2000	4.4	6.0	2000
	House (*n* = 15)	DF%	100	33	67	100
		Min (ng/g)	55	< LOD	<LOD	55
		Median (ng/g)	150	< LOD	0.8	160
		Max (ng/g)	670	22	5.1	670
		Mean (ng/g)	250	6.0	0.9	260
		SD	220	9.1	1.3	230
Saudi Arabia	House (*n* = 22)	DF%	100	100	100	100
		Min (ng/g)	140	0.2	0.9	150
		Median (ng/g)	440	1.2	2.9	440
		Max (ng/g)	1400	8.6	50	1400
		Mean (ng/g)	530	2.0	5.0	540
		SD	350	1.9	10	350
	Air conditioner (*n* = 3)	DF%	100	100	100	100
		Min (ng/g)	380	0.3	3.5	390
		Median (ng/g)	560	0.4	3.5	560
		Max (ng/g)	1500	1.2	14	1500
		Mean (ng/g)	810	0.7	7.0	820
		SD	590	0.5	6.0	600
	Car (*n* = 9)	DF%	100	100	100	100
		Min (ng/g)	120	0.6	1.4	120
		Median (ng/g)	190	1.5	2.7	190
		Max (ng/g)	280	2.8	6.6	290
		Mean (ng/g)	180	1.5	3.0	180
		SD	55	0.8	1.6	57
Vietnam	E-waste work shop (*n* = 7)	DF%	100	71	100	100
		Min (ng/g)	190	< LOD	0.4	200
		Median (ng/g)	290	0.3	2.0	290
		Max (ng/g)	870	1.2	4.6	880
		Mean (ng/g)	360	0.5	2.4	360
		SD	230	0.4	1.5	230
	E-waste living room (*n* = 8)	DF%	100	50	88	100
		Min (ng/g)	29	< LOD	< LOD	29

	Median (ng/g)	190	< LOD	0.6	190
	Max (ng/g)	11000	9.9	46	11000
	Mean (ng/g)	1700	4.3	7.4	1700
	SD	3800	4.5	17	3800
Office (*n* = 4)	DF%	100	25	75	100
	Min (ng/g)	17	< LOD	< LOD	17
	Median (ng/g)	160	< LOD	1.8	160
	Max (ng/g)	260	0.7	3.1	270
	Mean (ng/g)	150	0.7	2.3	150
	SD	100	0	1.5	100
Supermarket (*n* = 4)	DF%	100	75	100	100
	Min (ng/g)	61	< LOD	0.5	61
	Median (ng/g)	390	0.6	1.1	390
	Max (ng/g)	1100	1.6	2.4	1140
	Mean (ng/g)	490	1.0	1.3	490
	SD	500	0.6	0.9	500
Living room (*n* = 5)	DF%	100	60	100	100
	Min (ng/g)	17	< LOD	0.2	18
	Median (ng/g)	40	0.3	0.3	40
	Max (ng/g)	280	0.8	0.7	280
	Mean (ng/g)	97	0.4	0.4	98
	SD	110	0.3	0.2	110

Table S6 Parameters used for the estimation of human exposure to 1,3-diphenylguanidine (DPG) and derivatives through ingestion of indoor dust [1-3].

Country	Body wight (kg)					Exposure fraction (%)					Average dust ingestion (mg/day)				
	Infants	Toddlers	Children	Teenagers	Adults	Infants	Toddlers	Children	Teenagers	Adults	Infants	Toddlers	Children	Teenagers	Adults
Colombia	7	15	32	64	80	88	79	79	88	88	20	100	50	50	50
Greece	7	15	32	64	80	88	79	79	88	88	20	100	50	50	50
India	5	19	29	53	63	88	79	79	88	88	20	100	50	50	50
Japan	5	19	29	53	63	88	79	79	88	88	20	100	50	50	50
Kuwait	5	19	29	53	63	88	79	79	88	88	20	100	50	50	50
Pakistan	5	19	29	53	63	88	79	79	88	88	20	100	50	50	50
Romania	7	15	32	64	80	88	79	79	88	88	20	100	50	50	50
Saudi Arabia	5	19	29	53	63	88	79	79	88	88	20	100	50	50	50
South Korea	5	19	29	53	63	88	79	79	88	88	20	100	50	50	50
USA	7	15	32	64	80	88	79	79	88	88	20	100	50	50	50
Vietnam	5	19	29	53	63	88	79	79	88	88	20	100	50	50	50

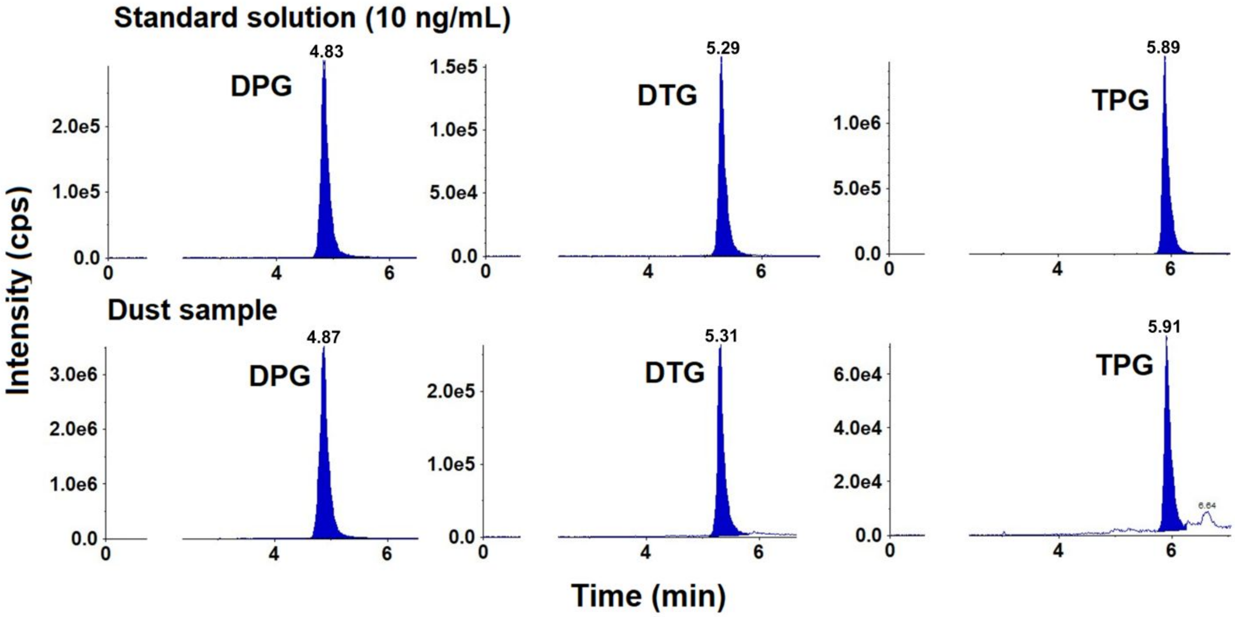

Figure S1 Representative chromatograms of 1,3-diphenylguanidine (DPG), 1,3-di-*o*-tolylguanidine (DTG), and 1,2,3-triphenylguanidine (TPG) in standard solution (10 ng/mL) and in dust samples. Injection volume was 2 μL.

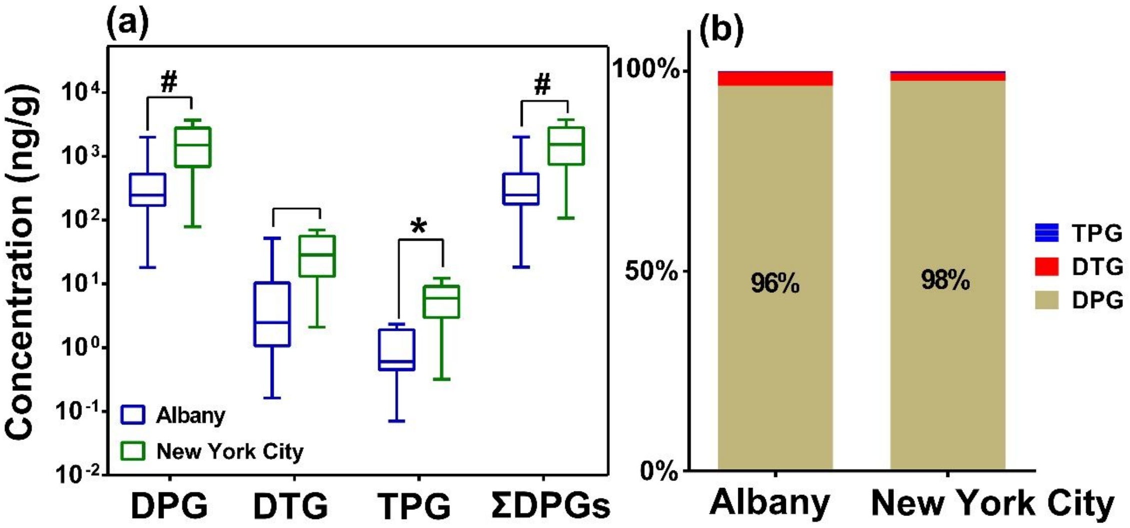

Figure S2 (a) Comparison of the concentrations of 1,3-diphenylguanidine (DPG), 1,3-di-*o*-tolylguanidine (DTG), 1,2,3-triphenylguanidine (TPG), and the sum of DPG and analogues (ΣDPGs) in house dust collected from Albany (n = 11) and New York City (n = 5), United States. The concentrations were compared using nonparametric test. # $p < 0.1$; * $p < 0.05$; p-values > 0.1 are not shown. (b) Stack distribution of DPG, DTG and TPG in house dust collected from Albany and New York City.

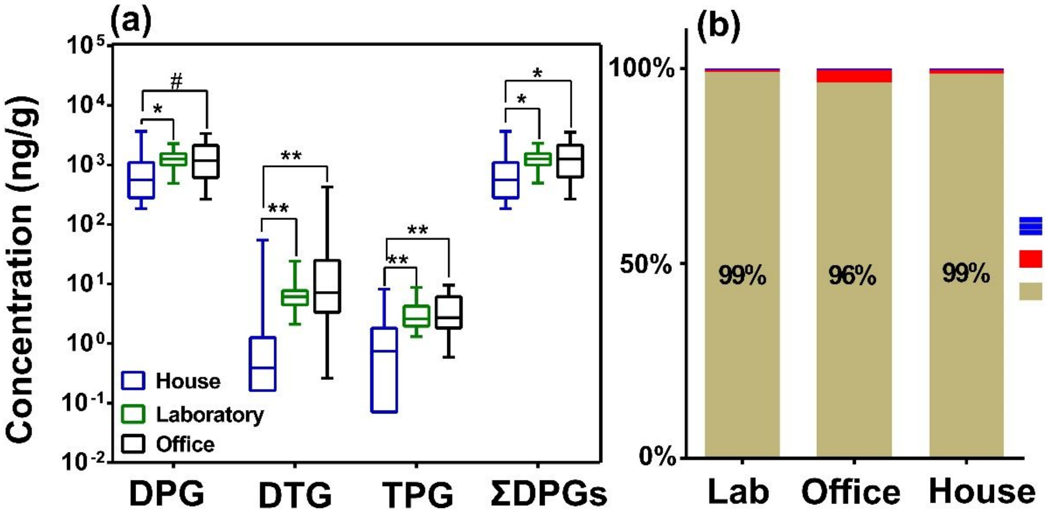

Figure S3 (a) Comparison of the concentrations of 1,3-diphenylguanidine (DPG), 1,3-di-*o*-tolylguanidine (DTG), 1,2,3-triphenylguanidine (TPG), and the sum of DPG and analogues (ΣDPGs) in dust collected from houses, laboratories, and offices of South Korea. The analyte concentrations among different microenvironments were compared using nonparametric test. # $p < 0.1$; * $p < 0.05$; ** $p < 0.01$; p-values > 0.1 are not shown. (b) Stack distribution of DPG, DTG and TPG in dust collected from houses, offices and laboratories in South Korea.

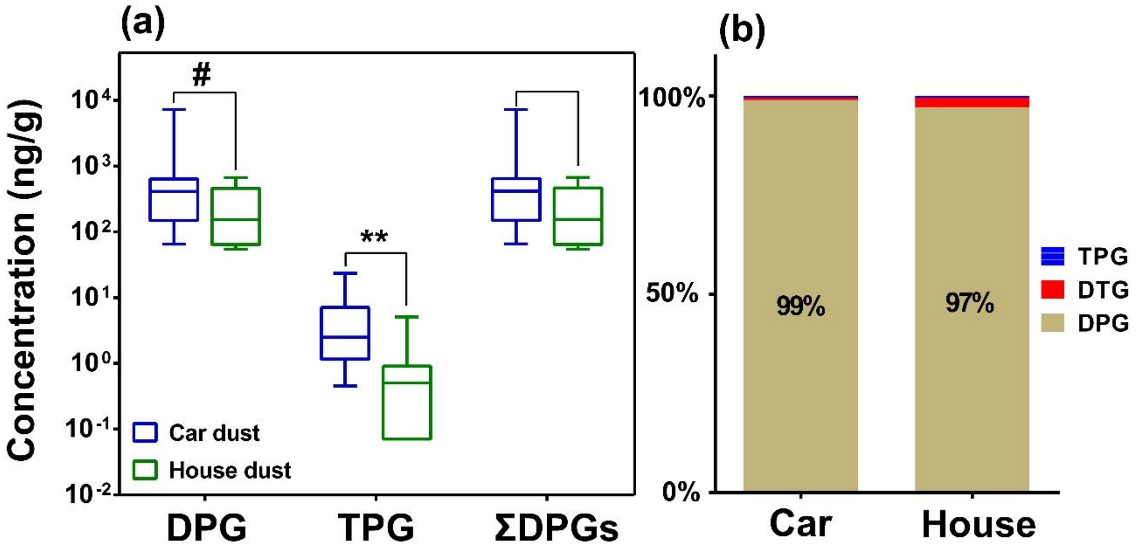

Figure S4 (a) Comparison of concentrations of 1,3-diphenylguanidine (DPG), 1,3-di-*o*-tolylguanidine (DTG), 1,2,3-triphenylguanidine (TPG), and the sum of DPG and analogues (ΣDPGs) collected from car and house in Kuwait. DTG was not included due to its low detection frequency (DF; 33%) in house dust. Nonparametric test was use for the comparison. # $p < 0.1$; ** $p < 0.01$; p-values > 0.1 are not shown. (b) Stack distribution of DPG, DTG and TPG in dust collected from cars and houses of Kuwait.

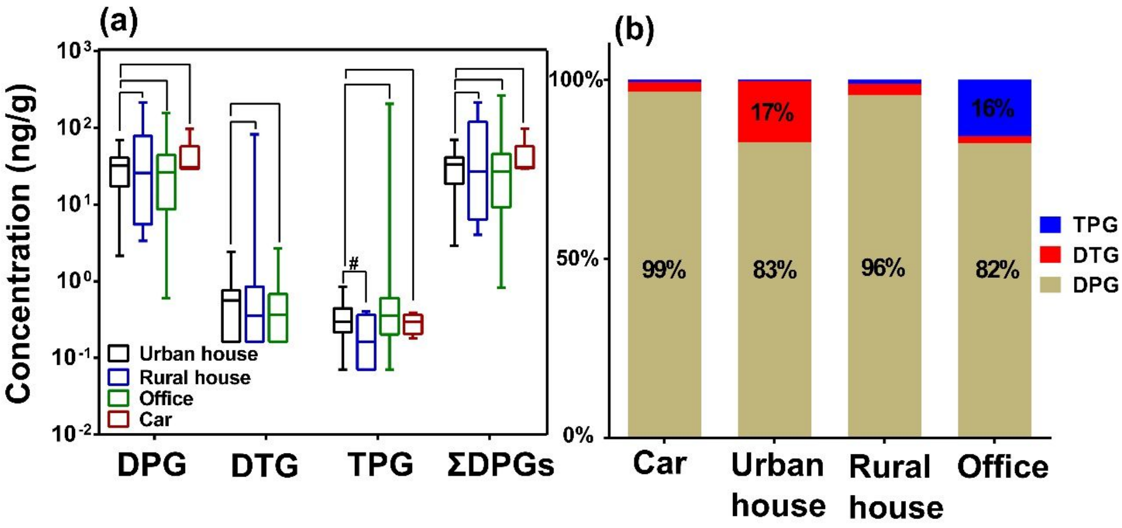

Figure S5 (a) Comparison of the concentrations of 1,3-diphenylguanidine (DPG), 1,3-di-*o*-tolylguanidine (DTG), 1,2,3-triphenylguanidine (TPG), and the sum of DPG and analogues (ΣDPGs) in dust from car, rural house, urban house and office of Pakistan. # $p < 0.1$; p-values > 0.1 are not shown. (b) Stack distribution of DPG, DTG and TPG in dust collected from cars, urban houses, rural houses, and offices of Pakistan.

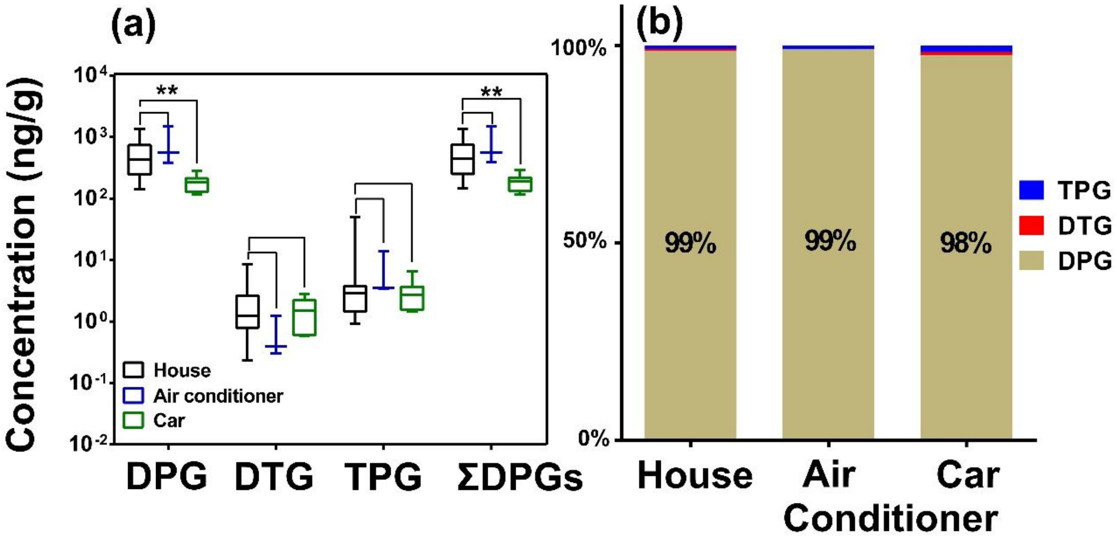

Figure S6 (a) Comparison of concentrations of 1,3-diphenylguanidine (DPG), 1,3-di-*o*-tolylguanidine (DTG), 1,2,3-triphenylguanidine (TPG), and the sum of DPG and analogues (ΣDPGs) collected from house, air conditioner, and car in Saudi Arabia. Nonparametric test was use for the comparison. ** $p < 0.01$; p-values > 0.1 are not shown. (b) Stack distribution of DPG, DTG and TPG in dust collected from houses, air conditioners and cars of Saudi Arabia.

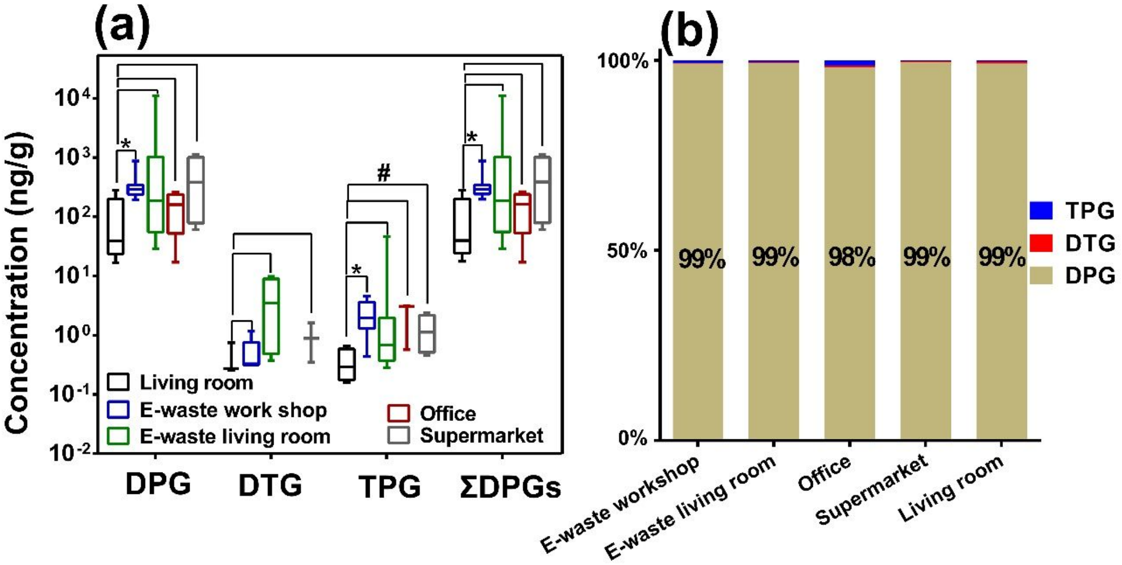

Figure S7 (a) Comparison of concentrations of 1,3-diphenylguanidine (DPG), 1,3-di-*o*-tolylguanidine (DTG), 1,2,3-triphenylguanidine (TPG), and the sum of DPG and analogues (ΣDPGs) collected from E-waste workshops and homes of Vietnam. Nonparametric test was use for the comparison. # $p < 0.1$; * $p < 0.05$; p-values > 0.1 are not shown. (b) Stack distribution of DPG, DTG and TPG in dust collected from houses (living rooms), e-waste workshops, e-waste living rooms, offices, and supermarkets of Vietnam.

References

1. EPA, Exposure Factors Handbook; United States Environmental Protection Agency: Washington, D. C., 2012. **2017**.

2. Liao, C.; Liu, F.; Guo, Y.; Moon, H. B.; Nakata, H.; Wu, Q.; Kannan, K., Occurrence of eight bisphenol analogues in indoor dust from the United States and several Asian countries: implications for human exposure. *Environ Sci Technol* **2012,** *46*, (16), 9138-45.

3. Zhu, H.; Kannan, K., Distribution Profiles of Melamine and Its Derivatives in Indoor Dust from 12 Countries and the Implications for Human Exposure. *Environ Sci Technol* **2018,** *52*, (21), 12801-12808.
